# 2447: Risk of adjacent segment breakdown at the cervico-thoracic junction: Where should we stop?

**DOI:** 10.1017/cts.2017.277

**Published:** 2018-05-10

**Authors:** Akachimere C. Uzosike, Venkata K. Byrapogu, Alim F. Ramji, Richard L. Skolasky, Brian J. Neuman

## Abstract

OBJECTIVES/SPECIFIC AIMS: Cervical fusion is commonly performed for the management of degenerative disc disease, which can cause spinal stenosis and radiculopathy. Adjacent segment disease (ASD) is an adverse postsurgical outcome experienced by some patients as new radiculopathy, stenosis, or other symptomatic sequelae. We sought to assess whether fusion extension past the cervicothoracic junction reduces the risk of distal ASD after multilevel fusions ending at C7-T3. METHODS/STUDY POPULATION: We retrospectively reviewed all first-time patients undergoing instrumented cervical fusion of at least 2 spinal levels and whose distal level of fusion ranged from C7-T3, at the Johns Hopkins Medical Institutions, from 1999 to 2013. The primary outcome was reoperation for distal ASD. Using multiple logistic regression, ANOVA, and χ^2^ analysis, we determined the odds of ASD due to age, gender, distal level of fusion, surgical approach (anterior, posterior, or combined), smoking status, and race. RESULTS/ANTICIPATED RESULTS: Of the 158 patients who met the selection criteria, the mean age was 58.7±13.8 years, and 95 (60.1%) were female. Ten patients (6.3%) underwent reoperation for ASD. Patients whose fusions ended at C7 were significantly more likely to develop ASD and undergo reoperation (70%, *p*=0.007) than those whose fusions ended at T1. There were no differences in age, proximal fusion level, smoking status, BMI, gender, and patient-reported race between the reoperation and non-reoperation groups. Following a multivariable analysis, extending the distal fusion to T1 was again found to be protective against reoperation (OR=0.07, *p*=0.020). DISCUSSION/SIGNIFICANCE OF IMPACT: Our study shows that for multilevel instrumented cervical fusions that terminate within the cervicothoracic junction, fusion distal to the C7 vertebra is associated with decreased odds of reoperation for symptomatic ASD. Therefore, this study provides clinical evidence that may help surgeons determine the optimal distal fusion segment for multilevel fusions ending at C7-T3.

